# Spinal mechanisms contributing to the development of pain hypersensitivity induced by sphingolipids in the rat

**DOI:** 10.1007/s43440-020-00207-x

**Published:** 2021-01-03

**Authors:** Hong Wei, Zuyue Chen, Ari Koivisto, Antti Pertovaara

**Affiliations:** 1 Department of Physiology, Faculty of Medicine, University of Helsinki, Haartmaninkatu 8, POB 63, 00140 Helsinki, Finland; 2 Research and Development, Orion Corporation, Orion Pharma, Tengströminkatu 8, POB 425, 20101 Turku, Finland

**Keywords:** Gap junction, Pain hypersensitivity, δ receptor, Sphingolipids, Spinal cord, TRPM3

## Abstract

**Background:**

Earlier studies show that endogenous sphingolipids can induce pain hypersensitivity, activation of spinal astrocytes, release of proinflammatory cytokines and activation of TRPM3 channel. Here we studied whether the development of pain hypersensitivity induced by sphingolipids in the spinal cord can be prevented by pharmacological inhibition of potential downstream mechanisms that we hypothesized to include TRPM3, σ_1_ and NMDA receptors, gap junctions and D-amino acid oxidase.

**Methods:**

Experiments were performed in adult male rats with a chronic intrathecal catheter for spinal drug administrations. Mechanical nociception was assessed with monofilaments and heat nociception with radiant heat. *N,N*-dimethylsphingosine (DMS) was administered to induce pain hypersensitivity. Ononetin, isosakuranetin, naringenin (TRPM3 antagonists), BD-1047 (σ_1_ receptor antagonist), carbenoxolone (a gap junction decoupler), MK-801 (NMDA receptor antagonist) and AS-057278 (inhibitor of D-amino acid oxidase, DAAO) were used to prevent the DMS-induced hypersensitivity, and pregnenolone sulphate (TRPM3 agonist) to recapitulate hypersensitivity.

**Results:**

DMS alone produced within 15 min a dose-related mechanical hypersensitivity that lasted at least 24 h, without effect on heat nociception. Preemptive treatments with ononetin, isosakuranetin, naringenin, BD-1047, carbenoxolone, MK-801 or AS-057278 attenuated the development of the DMS-induced hypersensitivity, but had no effects when administered alone. Pregnenolone sulphate (TRPM3 agonist) alone induced a dose-related mechanical hypersensitivity that was prevented by ononetin, isosakuranetin and naringenin.

**Conclusions:**

Among spinal pronociceptive mechanisms activated by DMS are TRPM3, gap junction coupling, the σ_1_ and NMDA receptors, and DAAO.

## Introduction

Dysregulation of sphingolipid metabolism has been associated with pathophysiological pain conditions [1]. Among spinal sphingolipid mechanisms contributing to pathophysiological pain is upregulation of an endogenous metabolite *N,N*-dimethylsphingosine (DMS) that can cause activation of astrocytes, release of proinflammatory cytokines and mechanical hypersensitivity [2]. In the central nervous system, damaged oligodendrocytes rather than astrocytes or neurons have been shown to be a source of DMS release and thereby an upstream mechanism of the DMS-induced pronociceptive effects [3].

Among spinal receptors that sphingolipids, including DMS, can activate is the transient receptor potential melastatin-3 channel (TRPM3) [4]. TRPM3 is a calcium-permeable ion channel that is expressed on various tissues, including nociceptive primary afferent neurons and the spinal cord (see for review [5]). Interestingly, oligodendrocytes that are a source of the endogenous DMS release express TRPM3 [5]. However, it is not yet known whether TRPM3 in the spinal cord might contribute to the pain hypersensitivity induced by DMS. Another receptor that DMS activates is the σ_1_ receptor [6]. In the spinal dorsal horn, σ_1_ receptors are expressed on astrocytes, where their activation is accompanied by astroglial release of D-serine, a co-agonist of the N-methyl D-aspartate (NMDA) receptor that promotes pain hypersensitivity [7, 8]. While it has been shown that DMS induces astrocyte activation that is accompanied by pain hypersensitivity [2], it is not yet clear whether astroglial σ_1_ receptors or NMDA receptors are involved in mediating the spinal DMS-induced pain hypersensitivity. Astrocyte activation has been associated also with upregulation of D-amino acid oxidase (DAAO), an astroglial enzyme that has a dual role in nociception [9]. On one hand, DAAO metabolizes D-serine and thereby attenuates pronociceptive drive by NMDA receptors. On the other hand, DAAO may facilitate pain by generating hydrogen peroxide that alone has pronociceptive actions [10] and that also drives the pronociceptive transient receptor potential ankyrin 1 (TRPA1) channel [11].

Here we studied the potential role of the spinal TRPM3 in the DMS-induced facilitation of pain behavior by assessing whether TRPM3 antagonists attenuate development of the DMS-induced pain hypersensitivity and whether pregnenolone sulphate (PS; TRPM3 agonist) recapitulates the DMS-induced hypersensitivity. Moreover, we determined whether spinal pretreatment with drugs inhibiting the astroglial σ_1_ receptor, astrocyte activation, the NMDA receptor or DAAO influences the development of the DMS-induced facilitation of pain behavior.

## Material and methods

### Experimental animals

The experiments were performed with 116 male Hannover-Wistar rats (220–260 g; Envigo Laboratories, Horst, The Netherlands) in Biomedicum Helsinki. The ethical committee for experimental animal studies of the State Provincial Office of Southern Finland (Permission # ESAVI/10218/04.10.07/2016) approved the experimental procedures. The experiments were performed according to the guidelines of European Communities Council Directive of 24 November 1986 (86/609/EEC). All efforts were made to minimize animal suffering, to reduce the number of animals used, and to utilize alternatives to in vivo techniques, if available. The animals were housed in polycarbonate cages with a deep layer of sawdust, two to three animals in each cage, in a thermostatically controlled room at 24.0 ± 0.5 °C. The room was artificially illuminated from 8.30 AM to 8.30 PM. The animals received commercial pelleted rat feed (CRM-P pellets, Special Diets Services, Witham, Essex, England) and tap water ad libitum.

### Techniques for drug administrations

For spinal administration of drugs, animals were installed an intrathecal (*it*; Intramedic PE-10, Becton Dickinson and Company, Sparks, MD, USA) under pentobarbital anesthesia at the dose of 60 mg/kg *ip* as described elsewhere [11]. After recovery from anesthesia, the correct placing of the catheter was verified by injecting lidocaine (4%, 7–10 μl followed by a 15 μl of saline for flushing) with a 50-μl Hamilton syringe (Hamilton Bonaduz AG, Bonaduz, Switzerland). Only rats that had no motor impairment prior to lidocaine injection but had a bilateral paralysis of hind limbs following it were included in the study. The volume of injections was 5 μl followed by a flush with 15 μl of saline.

In one control experiments, drugs were administered intraplantarly (*ipl*). *Ipl* injections were performed at a volume of 20 µl using using a BD Micro Fine insulin syringe with the 30G × 6.0 mm needle (Becton Dickinson and Company).

### Assessment of pain-related behavior

Monofilament-evoked limb withdrawal response to repetitive stimulation was used to assess mechanical pain behavior using calibrated monofilaments (North Coast Medical, Inc. Morgan Hill, CA, USA). Stimulus forces ranged from 2 to 26 g. Time course effects are reported only at the stimulus force of 6 g that induced no response, unless DMS had induced hypersensitivity. During testing, rats were on a grid inside a transparent box covering the grid. At each time point, the monofilament was applied to the plantar skin of the hind paw five times at a frequency of about 0.5 Hz. If the animal does not respond to monofilaments at all, its response rate is 0%, and if the animal withdraws its paw every time the filament is delivered, its response rate is 100%. An increase in the response rate represents pain hypersensitivity.

Heat nociception was assessed by determining withdrawal latency to noxious heat applied to the plantar skin using a radiant heat device (Plantar test model 7370, Ugo Basile, Varese, Italy). In the device, the rat is placed unrestrained on a glass panel and radiant heat is applied from a movable infrared generator below the glass panel to the hind paw until limb withdrawal that is detected automatically by a fiber optic sensor. The cut-off point was set at 15 s. Each measurement was replicated once at 1-min interval, and the average of the two successive measurements was used in further calculations.

### Drugs

*N,N*-dimethylsphingosine (DMS) [2], ononetin (TRPM3 antagonist [12]) and pregnenolone sulphate (PS; TRPM3 agonist [12]) were purchased from Tocris Biochemicals (Bristol, UK). Isosakuranetin, naringenin (TRPM3 antagonists), BD-1047 (σ_1_ receptor antagonist [6]), carbenoxolone (gap junction decoupler), MK-801 (NMDA receptor antagonist) and AS-057278 (DAAO inhibitor) were purchased from Sigma-Aldrich (St.Louis, MO, USA). The dose-range of DMS used in the present study (0.05–0.5 µg *it*) was chosen based on earlier studies [2, 13]. Doses of ononetin (100 µg *it*), isosakuranetin (10 µg *it*), naringenin (50 µg and 100 µg), PS (50, 150 and 500 ng *it*) and MK-801 (5 µg *it*) were chosen based on our preliminary experiments. BD-1047 was used at the dose of 0.5 µg *it* that has attenuated neuropathic pain hypersensitivity [7]. Carbenoxolone and AS-057278 were administered at the dose of 10 µg *it* that has attenuated pain hypersensitivity induced by sleep deprivation [14, 15]. Carbenoxolone, MK-801 and AS-057278 were dissolved in saline, BD-1047 in phosphate buffer, while DMS, ononetin and PS were dissolved in DMSO. Isosakuranetin was dissolved in Miglyol 812(Caesar & Loretz GmbH, Hilden, Germany) containing 0.1% DMSO. Phosphate buffer and 50% DMSO were used as vehicle controls.

### Course of the study

**Fig. 1:**
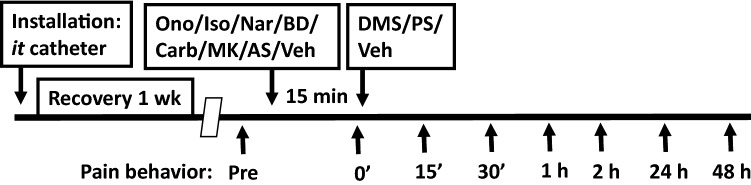
Time line for experimental procedures. *it* intrathecal, *Ono* ononetin (TRPM3 antagonist; 100 µg), *Iso* isosakuranetin (TRPM3 antagonist; 10 µg), *Nar* naringenin (TRPM3 antagonist; 50–100 µg), *BD* BD-1047 (σ receptor antagonist; 0.5 µg), *Carb* carbenoxolone (gap junction decoupler; 10 µg), *MK* MK-801 (NMDA receptor antagonist; 5.0 µg), *AS* AS-057278 (inhibitor of D-amino acid oxidase; 10 µg), *DMS* *N,N*-dimethylsphingosine (0.05–0.5 µg), *PS* pregnenolone sulphate (TRPM3 agonist; 0.05–0.5 µg)

Figure 1 shows the time line for experiments. Installation of *it* catheter under general anesthesia was followed by 1 week recovery. Habituation of the animals to the experimental conditions (1 hour for 3 days) was performed during the recovery period. When assessing the effects of ononetin, isosakuranetin, naringenin, BD-1047, carbenoxolone, MK-801 or AS-057278 pretreatments on the development of DMS-induced facilitation of pain, these compounds or vehicle (phosphate buffer) were administered 15 min before administration of DMS (0.5 µg) or vehicle (DMSO), and pain behavior was assessed up to 48 h as shown in Fig. 1. Additionally, in one experiment PS was administered alone (50, 150, or 500 ng), or PS was administered at the dose of 500 ng 15 min following administration of ononetin (100 µg), isosakuranetin (10 µg), or naringenin (50 or 100 µg). Mechanical and heat sensitivity was assessed before and at various time points up to 1 hour after administration of PS.

In one control group, DMS (0.5 µg) or vehicle (DMSO) was administered *ipl*, and pain behavior was assessed by stimulating the treated limb at various time points before and after *ipl* treatment.

The group size was 6–7, except that in the “vehicle followed by vehicle” groups *n* = 5. Since the effects of isosakuranetin and naringenin on DMS were tested several months later than other compounds, there was an additional (Vehicle +) DMS alone group (*n* = 6) to be tested in parallel with isosakuranetin and naringenin, the results of which were pooled with earlier tested (Vehicle +) DMS alone groups. The experiments were performed in blinded fashion. Each animal participated in 2–4 experiments. When testing the same animal more than once, the interval between tests was at least 1 week and the order of testing counterbalanced.

### Statistical analyses

Data were analyzed using *t*-test, one-way ANOVA or two-way mixed-design ANOVA followed by *t*-test with a Bonferroni correction for multiple comparisons. *p* < 0.05 was considered to represent a significant difference.

## Results

### Effect of DMS alone on pain behavior

**Fig. 2:**
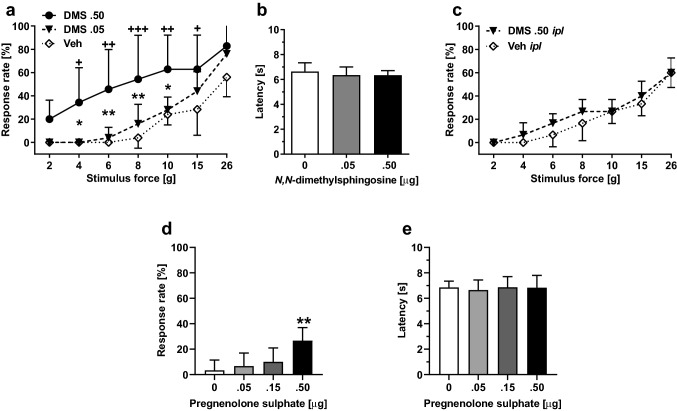
Effect by *N,N*-dimethylsphingosine (DMS) or pregnenolone sulphate (PS) alone on pain behavior. **a** Dose-related effect on mechanical sensitivity by *it* administration of DMS (at 60 min, which was the time point for the maximal effect). **b** Heat nociception following *it* administration of DMS. **c** Mechanical sensitivity following intraplantar administration of DMS. **d** Dose-related effect on mechanical sensitivity by *it* administration of PS (at 60 min tested at the force of 6 g). **e** Heat nociception following *it* administration of PS. In **a**, **c** and **d**, increases in response rate represent facilitation of mechanical sensitivity. The graphs show mean values. Error bars represent SD (in **a** and **b**, *n*_DMS .5_ = 7 and *n*_veh/DMS.05_ = 5, in **c**–**e**, *n* = 6). In **a**, ^+^/^++^/^+++^ indicate differences at corresponding time points between the DMS 0.5 µg and vehicle (Veh) groups, and */** indicate differences at corresponding time points between the DMS 0.5 µg and DMS 0.05 µg groups. In **d**, ** indicates difference to 0 µg (Vehicle) group. ^+^/*p < 0.05, ^++^/**p < 0.01, ^+++^*p* < 0.005 (*t*-test with a Bonferroni correction)

Spinal administration of DMS in healthy rats induced within 15-min facilitation of mechanically evoked pain behavior (mechanical hypersensitivity) that was maximal 60 min after DMS administration and disappeared 48 h after DMS administration. The DMS-induced mechanical hypersensitivity was dose-related (0.05–0.5 µg; *F*_2, 14_ = 5.81, *p* = 0.015; Fig. 2a). DMS at doses 0.05 µg or 0.5 µg *it* failed to influence heat nociception (*F*_2, 14_ = 0.48; Fig. 2b). At the currently used doses, *it* DMS did not evoke any obvious signs of ongoing pain.

To exclude the possibility that mechanical hypersensitivity induced by *it* administration of DMS was due to spread of DMS to the periphery, we assessed the effect of *ipl* administration of DMS (0.5 µg/20 µl) on pain behavior. DMS at a dose producing significant mechanical hypersensitivity effect following *it* administration did not have a significant effect on mechanically induced responses following *ipl* administration (*F*_1, 10_ = 1.23; Fig. 2c).

### Effect of pregnenolone sulphate alone on pain behavior

To study whether a spinally administered TRPM3 agonist recapitulates the DMS-induced hypersensitivity, pregnenolone sulphate (PS) was administered alone *it* at doses 0.5–0.50 µg. PS treatment induced mechanical pain hypersensitivity that was maximal within 15 min and lasted at least 1 hour (i.e., until the end of the observation period). The hypersensitivity effect was dose-related (main effect of dose tested at the stimulus force of 6 g: *F*_3, 20_ = 6.44, *p* = 0.003; Fig. 2d). PS *it* had no effect on heat nociception (*F*_3, 20_ = 0.10, *p* = 0. 96; Fig. 2e).

### Attempts to prevent the DMS-induced pain hypersensitivity with TRPM3 antagonists

**Fig. 3:**
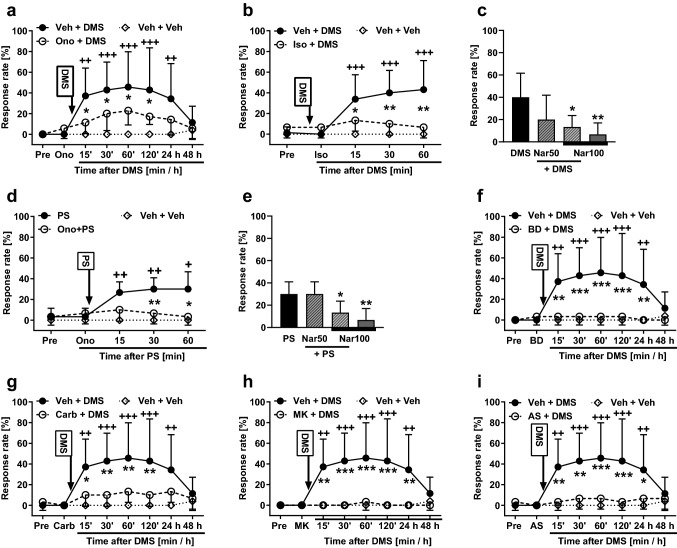
Effects of pretreatment with various compounds on the development of mechanical pain hypersensitivity induced by *N,N*-dimethylsphingosine (DMS; 0.5 µg *it*; **a**–**c** and **f**–**i**) or pregnenolone sulphate (PS; 0.5 µg *it*; **d** and **e**). **a** & **d** Pretreatment with ononetin (Ono; 100 µg). **b** Pretreatment with isosakuranetin (Iso; 10 µg). **c** & **e** Pretreatment with naringenin (Nar; 50 µg or 100 µg). **f** Pretreatment with BD-1047 (BD; 0.5 µg). **g** Pretreatment with carbenoxolone (Car; 10 µg). **h** Pretreatment with MK-801 (MK; 5.0 µg). **i** Pretreatment with AS-057278 (AS; 10 µg). Pre, baseline before drug treatments. All compounds were administered intrathecally. *Veh* vehicle. Pretreatments were given 15 min before DMS/PS that was administered at time point 0. Test stimulus force was 6 g. In **c** and **e**, time point of testing was 60 min after DMS or PS administration, respectively. Decreases in the response rate represent antihypersensitivity effect. The graphs show mean values. Error bars represent SD (*n* = 6, except that in **a** & **f**–**i** *n*_Veh+DMS_ = 7, in **b** & **c** *n*_(Veh+)DMS_ = 13, and in **a**–**b** & **f**–**i** *n*_veh+veh_ = 5). *p < 0.05, ^++^/**p < 0.01, ^+++^/***p < 0.005 (*t*-test with Bonferroni correction; reference for ^+^ is the value at the corresponding time point in the ‘Veh + Veh’ group. Reference for * is the value at the corresponding time point in the ‘Ono/Iso/BD/Carb/MK/AS + DMS/PS’ group, except that in c the reference is the DMS group and in e the PS group)

To study whether TRPM3 is involved in the development of mechanical hypersensitivity induced by DMS (0.5 µg *it*), the animals were pretreated 15 min earlier with ononetin, a TRPM3 antagonist (100 µg *it*). Spinal treatments (treatment as main factor: *F*_2, 16_ = 5.71, *p* = 0.013) and post-injection time (time as main factor: *F*_7, 112_ = 9.49, *p* < 0.0001) had significant effects on mechanical sensitivity that varied with the treatment group (interaction between main factors: *F*_14, 112_ = 4.16, *p* < 0.0001; Fig. 3a). Post hoc testing indicated that pretreatment with ononetin significantly attenuated the DMS-induced hypersensitivity for the first 120 min after DMS administration, but not anymore on the following day (Fig. 3a). To verify that the attenuation of the DMS-induced hypersensitivity by ononetin is indeed due to a block of TRPM3, the experiment was replicated using isosakuranetin (10 µg), another TRPM3 antagonist. Spinal treatments (treatment, including vehicle alone group, as main factor: *F*_2, 21_ = 9.41, *p* = 0.0012) had a significant effects on mechanical sensitivity (Fig. 3b). Post hoc testing indicated that the DMS-induced hypersensitivity effect was reduced by isosakuranetin at all time points studied (Fig. 3b). Pretreatment with naringenin, also a TRPM3 antagonist, produced a dose-related attenuation of the DMS-induced mechanical hypersensitivity (*F*_3,27_ = 5.86, *p* = 0.003; Fig. 3c). Post hoc testing indicated that naringenin pretreatment at the dose of 100 µg, but not at the dose of 50 µg, significantly attenuated the DMS-induced hypersensitivity effect (Fig. 3c).

To assess further the potential role of spinal TRPM3 in pain hypersensitivity, we attempted to prevent pain hypersensitivity induced by PS (a TRPM3 agonist; 0.5 µg *it*) using pretreatments with various TRPM3 antagonists. Ononetin at the dose of 100 µg *it* significantly attenuated PS-induced hypersensitivity (*F*_2, 14_ = 13.57, *p* = 0.0005; Fig. 3d). Naringenin attenuated PS-induced hypersensitivity in a dose-related fashion (*F*_3,20_ = 7.45, *p* = 0.0015; Fig. 3e); post hoc testing indicated that naringenin at the dose of 100 µg, but not at 50 µg, attenuated the PS-induced hypersensitivity effect (Fig. 3e). Also isosakuranetin (10 µg *it*) attenuated the PS-induced mechanical hypersensitivity effect (main effect of isosakuranetin on the PS-induced hypersensitivity: *F*_2, 14_ = 11.37, *p* = 0.0012; not shown).

### Attempts to prevent the DMS-induced pain hypersensitivity with other drugs

To study involvement of the σ_1_ receptor in the development of the DMS-induced mechanical hypersensitivity, the animals were administered *it* BD-1047, an antagonist of the σ_1_ receptor. In animals treated with 0.5 µg of BD-1047 followed by 0.5 µg of DMS, main effects of post-injection time (*F*_7, 105_ = 5.57, *p* < 0.0001), treatment (*F*_2, 15_ = 7.90, *p* = 0.0045) and their interaction (*F*_14, 105_ = 5.73, *p* < 0.0001) were significant (Fig. 3f). Post hoc testing indicated that the DMS-induced hypersensitivity was completely reversed by pretreatment with BD-1047 (Fig. 3f).

The role of gap junctions in the DMS-induced pain hypersensitivity was assessed by *it* pretreatment with carbenoxolone, a gap junction decoupler. In animals treated with 10 µg of carbenoxolone followed by 0.5 µg of DMS, main effects of post-injection time (*F*_7, 105_ = 7.32, *p* < 0.0001), treatment (*F*_2, 15_ = 6.01, *p* = 0.012) and their interaction (*F*_14, 105_ = 4.67, *p* < 0.0001) were significant (Fig. 3g). Post hoc testing indicated that the DMS-induced hypersensitivity was significantly reduced for the first 120 min by pretreatment with carbenoxolone, but not anymore on the following day (Fig. 3g).

To study the role of the NMDA receptor in the DMS-induced pain hypersensitivity, the animals were pretreated with MK-801, an NMDA receptor antagonist. In animals treated with 5 µg of MK-801, an NMDA receptor antagonist, followed by 0.5 µg of DMS, main effects of post-injection time (*F*_7, 105_ = 5.64, *p* < 0.0001), treatment (*F*_2, 15_ = 8.70, *p* = 0.003) and their interaction (*F*_14, 105_ = 6.12, *p* < 0.0001) were significant (Fig. 3h). Post hoc testing indicated that the DMS-induced pain hypersensitivity was completely reversed by pretreatment with MK-801 (Fig. 3h).

In animals treated with AS-057278 and DMS, post-injection time (F_7, 105_ = 5.983, *p* < 0.0001) and treatment (*F*_2, 15_ = 6.679, *p* = 0.0084) had significant main effects on hypersensitivity. Moreover, also interaction (*F*_14, 105_ = 5.646, *p* < 0.0001) between these main factors was significant (Fig. 3i). Post hoc testing indicated that pretreatment with AS-057278 significantly attenuated the development of the DMS-induced hypersensitivity up to 24 h (Fig. 3i).

Administration of ononetin alone (100 µg *it*, *n* = 6), isosakuranetin alone (10 µg), naringenin alone (50 µg or 100 µg *it*, *n* = 6), BD-1047 alone (0.5 µg, *n* = 5), carbenoxolone alone (10 µg, *n* = 6), MK-801 alone (5 µg, *n* = 5) or AS-057278 alone (10 µg, *n* = 6) had no effect on mechanical sensitivity (after all these drugs, the response rates overlapped with those in vehicle-treated animals; for the sake of clarity, data with these drugs alone are not shown in Fig. 3, except for naringenin alone at the dose of 100 µg in Figs. 3c, e).

## Discussion

In line with previous results, spinal administration of DMS induced in a dose-related fashion mechanical hypersensitivity [2, 13]. Hypersensitivity induced by single administration of DMS lasted up to 2 days, while in previous studies repetitive administration of DMS for 4 consecutive days induced hypersensitivity lasting for at least a week [2, 13]. Among novel results of the present study is that the DMS-induced pain hypersensitivity could be suppressed by spinal pretreatments with three different TRPM3 antagonists, σ_1_ receptor antagonist, gap junction decoupler, NMDA receptor antagonist or DAAO inhibitor. Moreover, spinally administered PS, a TRPM3 agonist, recapitulated the DMS-induced hypersensitivity in a TRPM3 antagonist-reversible fashion. Spinally administered DMS or PS selectively facilitated withdrawal responses evoked by mechanical but not thermal stimulation. Further, DMS or PS application did not evoke any apparent motor responses. Thus, current findings reflect actions on the sensory rather than motor system.

### Role of spinal TRPM3 in the DMS-induced pain hypersensitivity

Earlier studies indicate that TRPM3 expressed on peripheral terminals of nociceptive primary afferents nerve fibers is involved in transduction of heat [17]. The present results with three different TRPM3 antagonists (ononetin, isosakuranetin and naringenin) [12] and one TRPM3 agonist (PS), suggest that spinal TRPM3 plays a role in the induction of the DMS-induced mechanical hypersensitivity. Since peripheral terminals in a subpopulation of nociceptive nerve fibers express TRPM3 [17], also central terminals of nociceptive nerve fibers in the spinal dorsal horn are expected to express TRPM3. Thus, TRPM3 antagonists may have attenuated the development of the DMS-induced pain hypersensitivity due to action on TRPM3 expressed on central terminals of nociceptive nerve fibers by suppressing transmission to spinal pain-relay neurons that converge various types of inputs, including that from mechanoreceptors. Interestingly, it has been reported that TRPM3 is expressed on oligodendrocytes [5] that release DMS when exposed to inflammation or damage [3]. The potential role of oligodendrocytes in the DMS-induced pain hypersensitivity still remains to be studied.

### Other spinal mechanisms in the mediation of the DMS-induced pain hypersensitivity

Astrocytes are the most common cell type in the central nervous system. They can respond to neuronal signals, or astrocytes can be activated by various cytokines from microglia [16]. Astrocytes can also be activated by DMS released from injured oligodendrocytes [3]. Activated astrocytes can promote pain due to release of proinflammatory cytokines [16]. Astrocytes may promote pain also by forming gap junctions between astrocytes and neurons as well as surrounding astrocytes [18]. These gap junctions can be decoupled with carbenoxolone, which results in reduced pain hypersensitivity following, e.g., sleep deprivation [14] or nerve injury [19]. In the present study, spinal carbenoxolone treatment prevented the development of the DMS-induced pain hypersensitivity, which finding is in line with earlier results showing activation of astrocytes by DMS [2] and with the hypothesis that astrocytes have an important role in the DMS-induced facilitation of pain behavior. This hypothesis is further supported by attenuation of the DMS-induced hypersensitivity following blocking of the σ_1_ receptor, a receptor expressed on astrocytes [7, 8] and for which DMS is an agonist [6]. In the central nervous system, however, the σ_1_ receptor is also expressed on oligodendrocytes [20], a source of endogenous DMS release [3]. Thereby, an alternative or additional explanation for the antihypersensitivity of BD-1047 is that it prevented the spinal σ_1_ receptor-mediated release of endogenous DMS that promotes activation of astrocytes and thereby also release of proinflammatory cytokines [2].

Pretreatment with an NMDA receptor antagonist attenuated the development of the DMS-induced hypersensitivity. This finding is in line with the hypothesis that an increased astroglial release of D-serine that is a co-agonist of the pronociceptive NMDA receptor contributed to the DMS-induced pronociceptive action similarly as in neuropathic pain conditions [8].

In the central nervous system, DAAO is predominantly located in the astrocytes and it has a dual role in pain modulation [9]. The pronociceptive action of DAAO was predominant in the present study as indicated by the finding that pretreatment with a DAAO inhibitor suppressed the DMS-induced pain hypersensitivity. While increased DAAO activity is expected to reduce D-serine levels and thereby promote antinociception, other mechanisms, such as activation of astroglial serine racemase may have caused a net increase in the level and release of astroglial D-serine that provides an explanation for the contribution of NMDA receptors to the DMS-induced pain hypersensitivity. A plausible downstream mechanism for the DAAO-induced promotion of pain hypersensitivity is generation of hydrogen peroxide that alone is pronociceptive [10] and that additionally drives the pronociceptive TRPA1 channel [11].

Concerning other potential mechanisms, a recent study showed that among key mechanisms mediating pronociceptive actions of sphingosines is sphingosine-1-phosphate receptor expressed on astrocytes [21].

## Conclusions

The present behavioral results are in line with the hypothesis that among spinal mechanisms contributing to the DMS-induced pain hypersensitivity are TRPM3 channels, the σ_1_ receptor-mediated activation of astrocytes and/or oligodendrocytes, drive of NMDA receptors due to net increase and release of astroglial D-serine, and activation of astroglial DAAO that is expected to have its potential pronociceptive effect due to drive of hydrogen peroxide—TRPA1 pathway.

## Abbreviations

ANOVA: Analysis of variance
DAAO: D-amino acid oxidase
DMS: Dimethylsphingosine
DMSO: Dimethylsulphoxide
*Ipl*: Intraplantar
*It*: Intrathecal
NMDA: N-methyl-D-aspartase
PS: Pregnenolone sulphate
TRPA1: Transient receptor potential ankyrin 1
TRPM3: Transient receptor potential melastatin-3
